# A multisectoral approach to developing a national strategy for mental health and suicide prevention in Sweden

**DOI:** 10.1080/16549716.2026.2679336

**Published:** 2026-09-11

**Authors:** Lisa Becker, Olivia Biermann, Per-Olof Östergren, Sara Holsbrink, Jesper Sundewall

**Affiliations:** a Faculty of Medicine, Division of Social Medicine and Global Health, Lund University, Malmö, Sweden; b Department of Global Public Health, Karolinska Institutet, Solna, Sweden; c Public Health Agency of Sweden, Solna, Sweden; d HEARD, University of KwaZulu-Natal, Durban, South Africa

**Keywords:** Mental health, suicide prevention, health policy, public policy, multisectoral collaboration, Sweden

## Abstract

**Background:**

Mental ill-health is increasingly recognised as one of today’s major public health challenges. Many possible causes exist, distributed across all socio-political arenas. It is generally agreed that promotive, and preventive policies should be multisectoral, yet there is a lack of empirical studies concerning such endeavours. In a collaborative effort of 26 national governmental agencies, a proposal for a new national mental health and suicide prevention strategy was developed in Sweden.

**Objectives:**

To explore key stakeholders’ perceptions of the process for developing a new national strategy proposal.

**Methods:**

This is a qualitative study based on interviews with 25 representatives from 20 national governmental agencies and two external organisations. We used Directed Content Analysis and applied the Health Policy Triangle as analytical framework.

**Results:**

We found that the multisectoral collaboration led to agencies’ realisation of their limited knowledge about each other’s way of operating. The development process has been perceived as uncharacteristically long and loosely directed. Exploring contextual factors shed light on a need for better care, prevention, and promotion. For a successful implementation of the strategy, respondents demanded clear tasks from the government. Ultimately, respondents highlighted the collaborative implementation of the strategy over its content.

**Conclusions:**

The multisectoral developmental process was perceived as successful in convening various stakeholders to establish a basis for more collaborative work, particularly regarding the strategy’s implementation. The process did not only serve the purpose to develop a new strategy, but also allowed national agencies to learn about each other and how to collaborate going forward.

## Paper Context

**Main findings:** The multisectoral strategy development process successfully brought together 26 national agencies, revealing gaps in mutual understanding while fostering new cross-sector relationships that stakeholders view as essential for the future implementation of Sweden’s mental health and suicide prevention strategy.
**Added knowledge:** This study provides rare empirical insight into real-time multisectoral policy making in mental health, showing how collaboration processes, rather than strategy content alone, shape feasibility, shared ownership, and institutional readiness for implementation.
**Global health impact for policy and action:** The Swedish experience demonstrates that investing in multisectoral collaboration during policy development can strengthen national capacity to address complex public mental health challenges, offering actionable lessons for countries seeking to design and implement whole-of-government mental health strategies.

## Background

When it comes to mental health, all countries are developing countries. – Shekhar Saxena

Mental health is a fundamental aspect of human well-being and good mental health in the population is both an intrinsic value and a prerequisite for achieving other societal objectives. Over the past years, mental ill-health is increasingly being recognised as one of the major public health challenges of the 21st century, as illustrated by the adoption of relevant indicators in the United Nation’s Sustainable Development Goals. At the same time, the promotion of positive mental health has become a policy objective in itself [1]. In Sweden, while the majority of the population reports good self-rated health and good psychological well-being, psychological problems, such as stress, anxiety and sleep difficulties are at an all-times high among all age groups [2]. Due to the multifaceted causes of this public health problem as illustrated by the determinants of mental health, including individual, social, and structural determinants, policies in this area which aim to address the entire population should be multisectoral and include perspectives and shared perceptions of the underlying causal pathways.

A multisectoral approach, by definition, refers to the collaboration between various stakeholders (such as the government and civil society organisations (CSOs)) and sectors (e.g. the health, education or environment sectors) with the goal to achieve a policy outcome [3]. While there is broad agreement that multisector approaches are necessary to tackle today’s complex public health challenges, as resources can be pooled and common objectives can be identified, a lack of research analysing such endeavours exist [3–5]. Furthermore, the multisectoral developmental process of public mental health and suicide prevention policy remains understudied. Only few recent studies could be identified that conducted policy analysis which focus on the multisectoral developmental process of public mental health or suicide prevention policies [e.g 6–8]. Given that such policies are developed in a very heterogenous way, each case can provide unique insights to the broader topical literature.

Over the past decades, numerous national and regional policies on mental health and suicide prevention have been developed and updated in Sweden. Several key national policies and reforms within the area are depicted in Appendix A. From a large psychiatry reform in 1995, where many psychiatric services were transferred to social services [9], to the latest national mental health strategy which ran from 2016 to 2020 [10], Sweden has moved through various initiatives and policies aiming at strengthening patients’ rights and providing better care to those in need. Other key foci of the political instruments include the improved coordination between different levels of healthcare, agencies, and other parts of society, promoting evidence-based practices, and improving the accessibility and quality of mental health care services. Furthermore, the Swedish public health policy, introduced in 2018, has paved the way for a broad and holistic perspective on health and emphasises a strong focus on promotion and prevention [11].

In 2020, the Swedish government instructed the National Board of Health and Welfare and the Public Health Agency of Sweden to lead the work on submitting a suggestion for a new national strategy for mental health and suicide prevention in close collaboration with 24 other national agencies [12]. In the assignment, several key instructions were provided to guide the work, such as focusing on the overall public health policy framework and including various perspectives. For instance, a gender equality-, children’s rights-, youth-, and disability-perspective, clearly displaying a shift from traditional health care services and patient perspective to a more population-based perspective. The government emphasised that special attention should be put on promotion and prevention within all sectors of society, and societies’ most vulnerable groups should be highlighted. Three years later, in September 2023, the authorities proposed a ten-year strategy which includes four overarching goals and seven objectives, to be carried out in a broad cross-sectoral fashion. The four major goals of the proposal are improved mental health for the entire population, fewer lives lost to suicide, reduced inequalities in mental health, and reduced negative consequences due to psychiatric conditions [13].

### Purpose of the study

The scope of the strategy process under investigation is unique in many ways, such that for the first time, mental health and suicide prevention are merged into one national strategy. It is the biggest collaborative public policy development in the area of mental health to date, judging from directly involved governmental agencies and external stakeholders. Further, policy-making is not a straightforward process but can be messy, iterative, and non-linear [14]. For example, Lindblom described policy making for complex social problems, where attempts to improve mental health and well-being can certainly be regarded as a process of ‘muddling through’ a series of smaller policy options [15]. Therefore, analysing the unique development process of the proposal for a new national mental health and suicide prevention strategy in Sweden serves several purposes. First, the study can benefit future strategy revisions, as well as inform and support other countries in their development or revision of such a strategy. Second, understanding the developmental process of the strategy proposal might provide valuable insights relevant to the implementation process where many policy options will have to be considered alongside each other. Third, the study can shed light on the feasibility of such a broad intersectoral collaborative endeavour. Consequently, this qualitative interview study aims to identify and explore stakeholders’ perceptions of the development of a national mental health and suicide prevention strategy proposal.

## Methods

### Study design

This is a qualitative study based on semi-structured, in-depth interviews [16,17]. As means of data analysis, we chose Directed Content Analysis (DCA), in which theory guides the construction of codes [18].

### Theoretical framework

**Figure 1.**
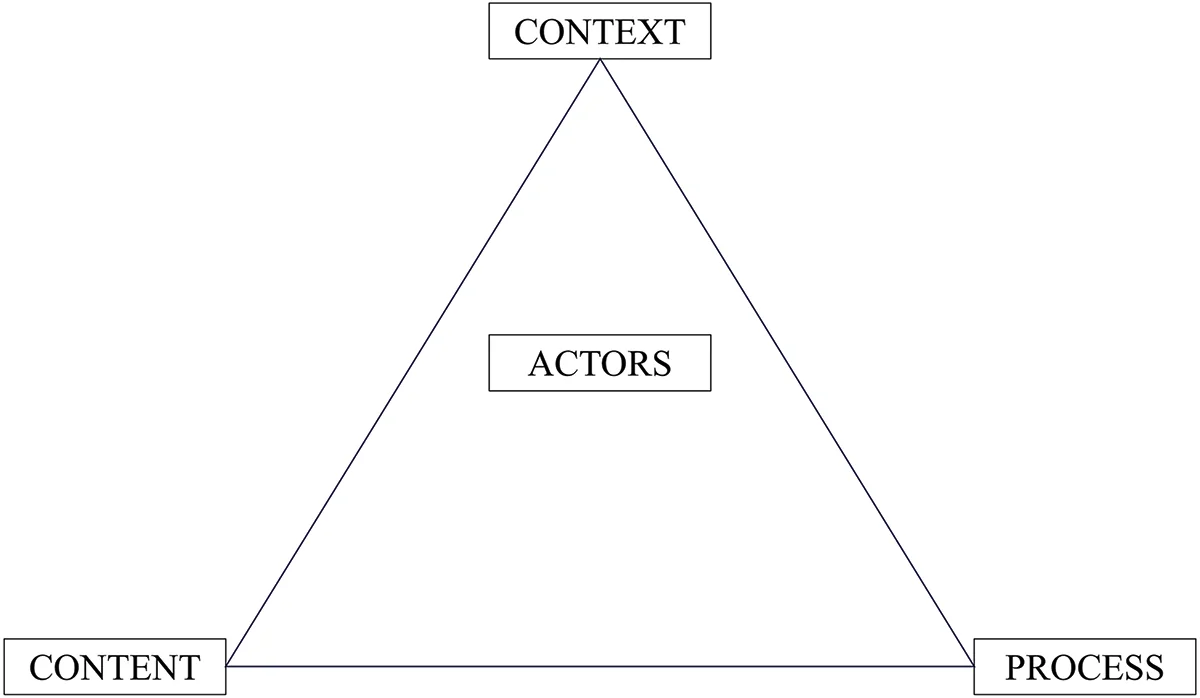
Health policy Triangle by Walt and Gilson, 1994 [19].

Guided by a pragmatic epistemological stance, we used the Health Policy Triangle (HPT) by Walt and Gilson [19] to analyse the content, context, process, and actors of an ongoing developmental process of a national strategy proposal [14]. A shortage of health policy analysis using the HPT framework exists [20], particularly in high-income countries. While several studies have applied the HPT framework to national and regional mental health policies [6,21,22], to the best of our knowledge, this is the first study to apply it to a national mental health and suicide prevention strategy proposal. Actors, which are at the centre of the HPT framework, include influential individuals, groups, organisations, companies, or the government, all of which may be interested in influencing the policy-making process. Context refers to the contextual factors that could affect health policy, such as economic, political, or social factors. Process refers to how a policy is made, including its development, formulation, implementation, and evaluation. Lastly, content relates to the policies’ objectives, guidelines, frameworks, etc [14]. An overview of the thematic framework is depicted in Figure 1. We applied the HPT to prospectively analyse for policy [14] due to the real-time documentation and analysis of concurrent health policy-making [23].

### Sampling of informants

Due to the study’s pre-defined population of interest, we used purposive sampling [17]. The concept of information power guided the initial and final sample size. We also considered data adequacy and continuously reflected on this during data collection and analysis. The study had a predefined and highly specific study population, i.e. representatives of national agencies formally involved in the strategy development. Therefore, the sampling aimed primarily to achieve variation across institutions and perspectives rather than thematic saturation in the conventional sense. Interviews were conducted across a wide range of agencies differing in mandate, size, and role in the process, and included both leading and peripheral actors. As data collection progressed, recurring patterns regarding perceptions of the process, actors, and contextual factors became evident, suggesting sufficient informational redundancy in relation to the study aim. At the same time, the inclusion of diverse agencies and roles ensured variation across perspectives, supporting the adequacy of the sample for an in-depth policy process analysis. Representatives from agencies, non-governmental- (NGOs), and civil society organisations (CSOs) involved in the process were contacted by email, outlining the purpose of the study, and inviting them to take part in an interview. Out of 26 governmental agencies, 23 representatives from 20 agencies accepted to be interviewed. Representatives of the six remaining agencies declined the interview due to either time constraints or without providing further reasons. Additionally, two non-governmental stakeholders (one CSO, and one member-based organisation) accepted the invite, amounting the sample size to 25. Due to several factors, such as the highly specific combination of actors for the study aim, the support of established theory, and strong interview dialogue, the sample size holds sufficient information power for the purpose of the study’s aims [24]. More information about participants’ affiliated agencies and organisations can be found in Appendix B.

### Data collection

Data collection took place in February and March 2023. Interview questions were mainly open-ended and targeted the predefined categories; actors, context, process, and content [18]. The first interview was treated as a pilot and resulted in minor changes to the introductory questions of the interview guide. Moreover, the interview guide for representatives from governmental agencies differed slightly from the interview guide for the two external stakeholders, as external organisations were not as closely integrated into the strategy development process and some questions were therefore deemed irrelevant. LB conducted all interviews online and in English, using Microsoft Teams software [25].

Interviews lasted between 11 and 75 minutes, with an average duration of 43 minutes. During one interview, three participants from the same governmental agency attended. On another instance, one participant from another agency was accompanied by a colleague who joined to support with English language should needs be, however ended up not having to be consulted. All remaining interviews were conducted one-on-one. After each interview, Microsoft Teams software [25] transcribed verbatim from interview recordings and LB checked each software-generated transcript against the interview recording.

### Data analysis

We applied Qualitative Content Analysis (QCA), following Hsieh and Shannon (2005) to analyse all collected data [18]. As a distinct approach to QCA, we chose DCA, which differs from a conventional QCA in that theory (in our case the HPT) guides the establishment of codes [18]. Therefore, DCA is also referred to as deductive content analysis [26]. Nonetheless, DCA can simultaneously be used to inductively identify new themes from data that does not fit pre-established codes, thereby enriching theory. Like other approaches to QCA, DCA is used to interpret meaning from text data content. Compared to conventional content analysis, DCA is guided by a more structured process [18]. Therefore, we analysed all data in several consecutive stages inspired by Kibiswa [27]. First, we identified concepts as codes using prior theory and subsequently established operational definitions for each code [28]. Second, once date acquisition was completed, LB read all transcribed interviews multiple times to ensure adequate immersion in the data. Third, LB coded all transcribed verbatim using NVivo software [29], re-reading all material interpretively and reflexively [30]. Simultaneously, codes were assigned to relevant meaning units using the prior established coding scheme. Additionally, all data that did not fit into existing codes’ definitions was coded inductively [18]. Fourth, once the coding process was completed, we made connections, which were interpreted, and drew conclusions. In this final stage of the analysis, in an effort to make sense of the data, we built logical chains of evidence, which we interpreted both at manifest and latent content levels [18]. Consequently, a new theme, ‘implementation’, emerged inductively from the coded data [26].

### Trustworthiness

To increase the study’s trustworthiness, thick descriptions of the entire research process are provided in the form of extensive contextual descriptions, the inclusion of tables, appendices, and quotations, to complement the interpretations made and the conclusions drawn [27]. The context provided will help the reader to make their own assessment of the study’s credibility, transferability, dependability, and confirmability [31]. Beyond the use of thick descriptions, trustworthiness was strengthened through multiple strategies: the systematic use of extensive field notes during interviews and analysis, data source triangulation via interviews with diverse stakeholder groups (governmental agencies and external organisations), and iterative peer debriefing and member checking among the authors across the analysis process.

## Results

**Figure 2.**
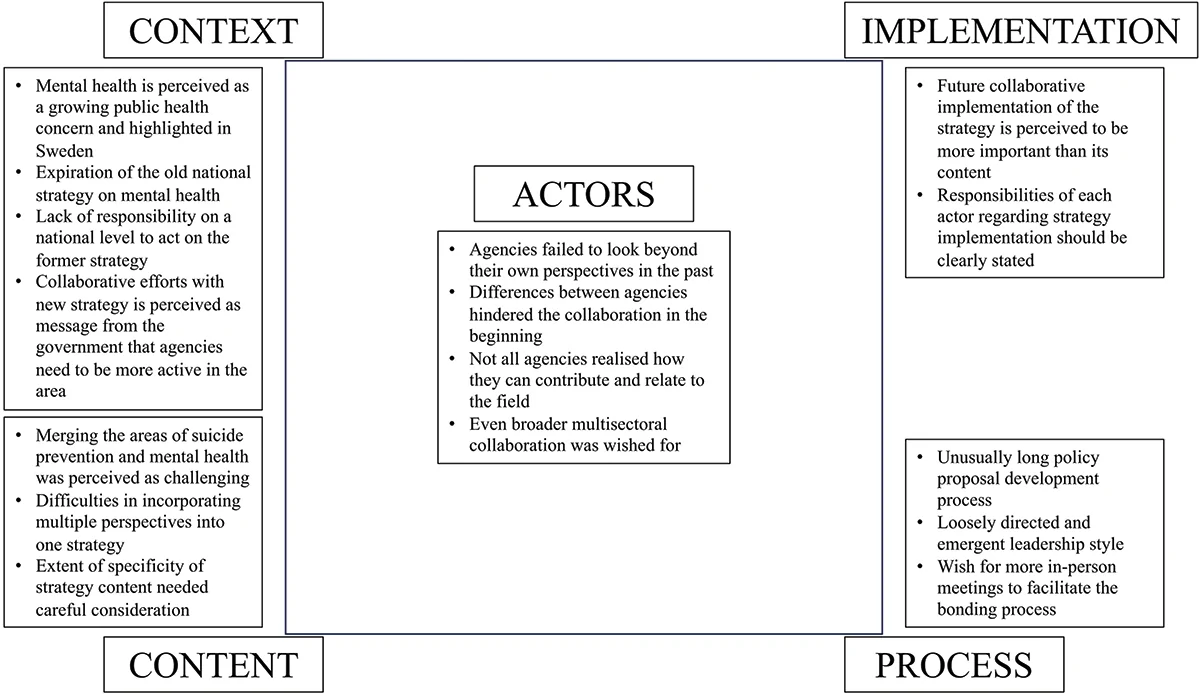
Main study findings structured according to the expanded health policy Triangle framework, including implementation as a main theme that emerged throughout data analysis, however is an integral part of the process component.

Findings are clustered into the four inherent components of the HPT framework and the newly identified theme implementation. The study’s main findings alongside the expanded HPT framework can be found in Figure 2.

### Content

Regarding the strategy’s content, many discussions revolved around how to balance the space and priority given to different areas. It is the first time in Sweden that policy in mental health and suicide prevention is merged, and respondents perceived it as difficult to decide how much space each of these areas should receive. Actors that are active in suicide prevention, from both governmental agencies and organisations, feared that this area might be overlooked in the broader field of mental health and therefore risks inadequate attention. Nevertheless, the ambition was stated that suicide prevention should be given sufficient space within the strategy while trying to keep both areas together and not splitting them fully, as they are highly interrelated.

Some actors that work specifically with suicide prevention were concerned that the wider and the bigger area of mental health will take all the focus from suicide prevention. But our ambition is to give suicide prevention a clear space in the strategy, while also trying to keep both areas together as one and not split them. (P-6)

Respondents further reported difficulties in incorporating all the different perspectives into the strategy. One of the challenges was that while some perspectives should be considered throughout the strategy, such as the gender-equality perspective, others should be mentioned by themselves, such as the Sami perspective. Additionally, some respondents reflected that the gerontological perspective was often overlooked and forgotten during the conception of the content, despite older men being a high-risk group for dying by suicide in Sweden. Considering the overall direction of the strategy proposal, respondents deemed it especially important to address mental health holistically. They argued that it is essential to move away from only considering mental health systems and instead shifting the focus to prevention and promotion.

Another big discussion among respondents revolved around finding the right amount of specificity for the content of the strategy proposal. Walking the thin line of neither being ‘too narrow’ nor ‘too broad’ was repeatedly mentioned to be one of the biggest challenges in this process. While some agencies argued for a need for concrete changes, for example, in legislation concerning the right to share sensitive health information between sectors, others wanted the strategy proposal to be a broad framework that can be applied to all sectors of society. Respondents mentioned that being broad and relatively abstract makes it easier for all parties to agree on the content. Nevertheless, others noted that being too broad comes with challenges, such as forgetting about minority groups. Additionally, stakeholders expressed their worries that the strategy risks becoming fragmented when many societal sectors should be given space. Overall, it was stressed that the ambition is to create content that is relevant for all 26 governmental agencies and that the strategy should be easy to work with for all stakeholders, transcending into other parts of society, such as CSOs.

It’s a challenging process to write and decide on the content of the strategy, and there are so many sectors in society that should be given space. (P-5)

### Actors

The constellation of actors that were commissioned with the development of the strategy proposal has been a unique assembly of national governmental agencies and external organisations. While the two lead agencies and the remaining 24 governmental agencies were forming the core group of actors, many more stakeholders were involved including NGOs, CSOs, international organisations, or member-based organisations. Several respondents from governmental agencies mentioned that the strategy development process made them realise how little they knew about the other national agencies’ work and the way each one of them operates. Reportedly, the agencies failed to look beyond their own perspectives in the past.

We realised that we know quite little of each other’s work. We’re quite narrow-minded. We think a lot about our agencies and our perspective. But we are not very good at trying to have a broader perspective … So that’s been like an awakening. (P-18)

Differences between the agencies hindered the collaboration in the beginning. Some smaller-sized agencies reported feeling more flexible and freer in the way they could collaborate, while structural hurdles of bigger agencies limited their flexibility. Nonetheless, most stakeholders viewed this collaboration as a major opportunity to get to know the other agencies and their way of operating better and understood this assignment as a fundament for more collaborative work to come.

I think it’s good to see each other’s backyards. (P-12)

Several agencies struggled to see how their work relates to the field of mental health and suicide prevention, and while some were eager to find their place, others showed little motivation to engage in the area more actively. Nonetheless, the lead-agencies described the overall ambition to keep as many stakeholders as possible on board and help them understand how they can contribute to the strategy and area.

I think there are some agencies that contributed a lot, and others that didn’t have so much to contribute with. I don’t feel that we had that much to contribute with. (P-1)

It’s really important that every agency does their part. Because it’s a really important question for the whole society. (P-10)

Despite efforts to reach out to and consult with stakeholders at different levels, most respondents believed that more collaboration with a broader spectrum of actors would have been favourable. Several respondents mentioned that some stakeholders should have been included in the strategy process to a greater extent. Among those was SALAR, which concurrently worked on a regional strategy on mental health. Respondents also mentioned that CSOs should be involved more closely due to their critical knowledge and expertise in the field, a more practical understanding of the problems, and their important role during the strategy’s implementation. Additionally, several respondents commented that the Swedish Public Employment Service was not included in the process, although they are an important stakeholder supporting people with mental health conditions to re-enter the labour market. Lastly, some respondents noted that international actors, such as the World Health Organisation, should have been included to a larger extent.

### Process

Respondents perceived the process as unusually long compared to the development of other national policies. Some respondents expressed criticism that too much time was spent on bringing forward a strategy and getting to know each other, while the focus should be on urgently needed action.

I think that … they’ve spent longer time getting along and getting to know each other and just hearing each other’s different perspective as agencies than actually working together. (P-24)

The strategy development followed a multi-stage, iterative process coordinated by the two lead agencies. Initially, participating governmental agencies were clustered into smaller working groups based on broad areas of expertise. These groups met primarily through digital meetings due to COVID-19 restrictions, which characterised the early phase of the assignment.

In parallel, all governmental agencies and selected external stakeholders were tasked with conducting agency-specific analyses during the first year of the process. These analyses focused on perceived priorities within mental health and suicide prevention, each agency’s role and mandate in the field, and key challenges from their institutional perspective. The results were submitted to the lead agencies and synthesised into a joint mid-term report, which was presented to the government.

In the subsequent phase, the lead agencies used this material to draft a national strategy framework, including overarching goals, priority areas, and proposed indicators for follow-up. This drafting phase involved repeated plenary meetings with all governmental agencies, during which draft versions of the strategy were presented, discussed, and revised based on collective feedback. Agencies primarily contributed input related to their mandates, while also commenting on the overall coherence and feasibility of the strategy.

Alongside these plenary meetings, the lead agencies held targeted dialogues with external stakeholders, including civil society and member-based organisations, to obtain additional perspectives and address identified gaps. During the final months of the assignment, near-final drafts of the strategy were presented to external stakeholders for feedback, which informed the concluding revisions prior to submission.

### Context

Exploring stakeholders’ perceptions of the contextual factors that surround the development of the strategy proposal shed light on societal developments and past political efforts in the field. All respondents shared an overall perception that mental health is a growing public health concern which is highlighted in Swedish society and politics. While mental health and suicide prevention have arguably been neglected in the past, respondents perceived the area to have gained increased political attention over the past two decades. This is reflected in that mental health and suicide are frequently debated in Swedish media, contributing to an open societal discourse. Respondents agreed that mental illness causes individual suffering and high financial costs for society and there is a clear need for better care and prevention in Sweden. Moreover, respondents reported a general lack of resources and a mismatch between the attribution of healthcare funding towards mental and physical health, with physical health receiving the majority of financial resources.

Mental health is very much highlighted in society and in politics, and everyone agrees that it’s a big and increasing problem that needs to be in focus. (P-5)

Many stakeholders indicated that the development of a new strategy proposal takes place in the context of the expiration of the previous national strategy on mental health, running from 2016 to 2020. This former national mental health strategy marked a big turn in the perception of mental health governance in Sweden. Before 2016, efforts in mental health had focused on psychiatric care and particularly vulnerable groups, such as children, youth, and people with severe mental health conditions. With the introduction of the previous strategy, a broader approach to tackle the field of mental health was introduced, addressing many sectors of society. While respondents considered the content of the former strategy still relevant, problems existed regarding its implementation. There were no follow-up systems that could capture changes in relevant indicators, and at national level no actor had overall responsibility for implementing the strategy, leading to fragmentation. Stakeholders noted similar shortcomings of suicide prevention policies. The lack of responsibility at national level concerning the former mental health strategy resulted in a shift of responsibilities towards the regions and municipalities, which cannot be mandated to work with national policies as they are soft governance tools and therefore only constitute guiding frameworks without legislative power. Ultimately, the decentralised system in Sweden, where healthcare is governed at the regional level and social services are the responsibility of municipalities, was highlighted as a challenge by some respondents, as it can complicate cross-sectoral work.

Participants mentioned that the new mental health and suicide prevention strategy aims to maintain a broad focus while considering what it means for the entire society to contribute to improving mental health. All stakeholders agreed that more collaboration is needed between governmental agencies, as agencies often work within silos. Respondents elaborated that from 2015 to 2018, the Swedish government appointed an independent investigation to review the area of mental health, which resulted in a proposal that intersectoral efforts are needed to develop a new national strategy. Therefore, respondents perceive the development of a new strategy proposal as a clear message from the Swedish government that governmental agencies need to be more active and involved in this area and should contribute with their individual and combined expertise and resources.

### Implementation

Many respondents reported their perceived importance of *how* the strategy will be implemented and highlighted this over the content of the strategy. While the strategy proposal provides a framework for mental health, priorities are likely to change over time as there are many open questions prevailing about what needs to be done in the area.

What we do together in the future will be the most important. So, all this text, I mean, it’s good, and we need to see the picture, of course. But what we do together [is more important]. (P-2)

There are also expectations on guidance from the government during the implementation of the strategy. Respondents noted that the government should assign actors with specific tasks, clearly defining what needs to be done and who should do it. Several participants feared that without clearly assigned tasks, history could repeat itself, and the strategy would end up looking good on paper, but no actor would feel responsible for its implementation. Respondents added that with clear guidance from the government, agencies will prioritise the implementation. Additionally, funding should be attached to work with the strategy for smaller agencies, since some agencies don’t have earmarked funding for mental health and suicide prevention.

The strategy needs to be clear about who should be aiming at doing what. Otherwise, you leave it; it’s just a piece of paper. People can read it, but they don’t … want to do anything. Someone needs to take the lead and make sure it happens as well. Otherwise, I think it’s pretty hopeless. (P-1)

## Discussion

This qualitative study sheds light on key stakeholders’ perceptions of the development process of a new national mental health and suicide prevention strategy proposal in Sweden. Our findings indicate that the specificity of the content of a national mental health and suicide prevention strategy needs to be carefully balanced to ensure that it is neither too broad nor too detailed. The development of the strategy proposal brought together a large set of actors, including governmental agencies and various organisations, in a way that is considered unique for public policy development in Sweden. Through this process, the participating agencies realised how limited their understanding of each other’s work was. Furthermore, the process was perceived as long and loosely directed, both from the government and from the leading agencies, which differs from other national policy developmental processes in Sweden. Our analysis of the contextual factors depicted prior political efforts in the area, showcased a strong societal discourse, and a recognised need for better care, prevention, and promotion. Lastly, participants largely agreed that clear instructions from the government are needed for the strategy’s successful implementation.

Arguably, the most outstanding feature of this strategy proposal development lies in its multisectoral approach, convening 26 national agencies alongside other stakeholders, such as NGOs or CSOs. There are several advantages to multisectoral approaches, such as addressing the area of mental health and suicide prevention in a focused way through pooling resources, thereby avoiding the duplication of inputs [3]. Further, a prerequisite to the policy implementation is political will, particularly from the Swedish government. Their mandate provided at policy level facilitated the successful multisectoral coordination. As reflected by many representatives of governmental agencies, the collaboration led to the development of institutional mechanisms which is deemed essential for the standardisation process of intersectoral coordination [3].

Tackling mental ill-health and promoting good mental health can be described as a ‘wicked problem’, i.e. a problem that is characterised by complex causality, multiple and often competing understandings, contested goals, a lack of definitive solutions, unintended consequences, and an incomplete evidence base [32], thereby posing threats to contemporary governance and policy-making [33]. The underlying social determinants of mental health add to the complexity of the area and complicate political efforts to address the problem. According to Head and Alford, collaborative efforts are one primary strategy for addressing such complex societal issues [33]. Next to taking a broader approach in thinking about societal problems, ways to address them, and new leadership propositions, cooperative approaches can be used as central instruments to tackle wicked problems. Head and Alford argue that a functioning collaboration between many stakeholders can increase the likelihood of better understanding the underlying causes of a wicked problem and finding or agreeing upon possible solutions, resonating with our findings [33]. Implementation of solutions to complex problems can be facilitated through collaboration as it enables shared contributions and coordinated actions [33].

The idea of ‘joining forces’ and going about an intersectoral approach to implement health policy is not new, dating back to the 1970s [14]. Reports such as the Ottawa Charter for health promotion and the concept of Health in All Policies (HiAP), are a few examples of similar aspirations [34,35]. Nonetheless, policy implementation has almost equally long been identified as a challenging endeavour [36]. Oftentimes, a discrepancy exists between what a policy ought to achieve and what is achieved in practice; the ‘policy-implementation gap’ [14,37]. While increased efforts try to fill this gap, progress remains patchy [14]. Hudson et al. argue that inadequate collaborative policy-making is one of four broad contributors leading to policy failure [38]. Accordingly, failure to establish a common ground is one of the key reasons for implementation difficulties [38]. Another major threat to policy implementation is that policymakers may not actually want to see a specific policy implemented [39]. Policy implementation often lies in the hands of various stakeholders, which might not align with their interests and thus use different techniques to resist policy change [19]. Yet, our results indicate no conflict of interest regarding the policy’s objective and aim, as improving the area of mental health and suicide prevention has been acknowledged by all study participants. Nonetheless, some agencies failed to see their own responsibility in contributing to better mental health and suicide prevention, thereby jeopardising a successful implementation. Overall, however, findings from this study indicate good potential for a successful implementation, as the policy was developed in a collaborative effort, and stakeholders repeatedly indicated the importance of a collaborative implementation.

Both the COVID-19 pandemic’s influence on the rise of mental health issues and the debated effects of social media on young people’s mental health are frequently attributed to the stark mental health decline globally in recent years, in the academic and grey literature as well as popular media. Interestingly, however, these two major contextual factors of the current ‘mental health crisis’ particularly among children and youth, have been mostly absent in participants’ reflections on the overall context. The pandemic severely impacted people’s lives and resulted in a surge in mental illness as several life stressors were added at once, such as potential health impacts of the virus, public health measures taken to contain the spread of the virus and financial insecurity [40]. The prevalence of depression, anxiety, and substance abuse rose, increasing the demand for mental health services. Simultaneously, the pandemic has caused major disruptions to the provision of these services [41]. In the pandemic’s aftermath social media is taking centre stage as a major driver for young people’s decreasing mental health, supported by an increasing body of evidence [42,43]. However, contradictory findings showcase both negative and positive effects of social media use and cause and effect are difficult to determine [44,45]. To counteract this worrisome development policymakers globally are proposing and adopting legislation such as setting age restrictions on the use of social media platforms.

Our results further highlighted that representatives from governmental agencies want to receive specific instructions from the government to implement the strategy. Clearly defined roles and responsibilities have been highlighted as a prerequisite to the well-functioning coordination of multisectoral collaborations which should be established before implementation [3]. Work by Rasanathan et al. emphasises that effective multisectoral action for health is fundamentally a governance challenge, requiring mechanisms to manage incentives, roles, and collaboration across sectors, and clear leadership to align diverse actors around shared objectives. These arguments have been advanced in analyses of multisectoral action in low- and middle-income settings, highlighting that implementation processes, accountability structures, and political economy dynamics are central to whether collaborative policies achieve impact [46]. The former Swedish national mental health strategy has been criticised for inadequately tackling structural factors which reportedly influence mental health [47], such as social determinants of health, including poverty [48]. Responsibility to address structural factors was shifted towards the regions, for which they neither had a mandate nor the means [47]. At a national governmental level, agencies reportedly did not feel responsible for working with the former strategy. To avoid renewed confusion of responsibilities, the desire for clear governmental assignments within the framework of the new strategy were stated. Additionally, stakeholders clearly voiced a need for funding attached to assignments to work with the strategy, as one major obstacle to successful health policy implementation are insufficient financial resources [39].

Finally, a major finding from our study pertains to the importance participants assigned to the implementation of the adopted strategy. Highlighting policy implementation over policy content strongly aligns with Lindblom’s idea of ‘muddling through’ [15]. As a steadfast critique of the rational planning model, Lindblom argues that particularly for complex problems the rational-comprehensive method to decision making is not achievable, particularly considering limited available funds. Henceforth the dismissal of the importance of the content by most study participants reflects a realistic approach to achieve opportunistic policy goals in the area of mental health and suicide prevention. Participants also mentioned that priorities within the area are likely to shift over time, in line with Lindblom’s ‘succession of comparisons’, stating that policy is made over and over again approximating some desired end state which itself might change over time [15].

### Limitations of the study

Several methodological limitations warrant consideration. All interviews were conducted online, in English and by a single interviewer. First, while online interviews have advantages, such as mitigating the distance of space [49] and allowing for equal rapport building between interviewer and interviewee as in face-to-face interviews [50], they also have shortcomings. One relevant shortcoming is that online interview formats hinder the interviewer in perceiving and interpreting the participants body language [51]. Second, interviews were conducted in English, which was not the first language for most participants. This may have affected participants’ ability to articulate nuanced perspectives. While participants were offered clarification and support during interviews, some degree of meaning loss or simplification cannot be ruled out. Third, all interviews were conducted by a single interviewer. While this enhanced consistency in interview conduct and facilitated the development of in-depth contextual understanding of the policy process, it might also have limited perspectival diversity and introduced interviewer-specific biases in data generation. To counteract this, the authors used reflexive practices and peer debriefing throughout the analysis [52]. Lastly, at the time point of data collection, the strategy’s content was still work in progress. Therefore, participants could not share their reflections about the final content of the strategy proposal or how the entire project was perceived after its completion.

## Conclusions

The development process of the Swedish mental health and suicide prevention strategy proposal helped fostering multisectoral collaboration, according to study participants, which is expected to positively impact the future strategy’s implementation. The implementation process is expected to require strong leadership and clear assignments from the government. Findings outlined in this study may support policy development across sectors, which need to be adopted to country contexts. Future research should follow developments and evaluate the strategy’s implementation to determine if this comprehensive approach to public policy development can successfully address complex and intersectoral problems in Sweden and contribute to improved mental health and well-being for the population.

## Supplementary Material

COREQ_Checklist.pdf

Supplementary data 28 april 2026.docx

## Data Availability

Data will be made available upon reasonable request.

## Abbreviations

**CSO:** Civil Society Organisation

**DCA:** Directed Content Analysis

**HPT:** Health Policy Triangle

**NGO:** Non-governmental Organisation

**SALAR:** Swedish Association of Local Authorities and Regions

**QCA:** Qualitative Content Analyses

**HiAP:** Health in All Policies
