# Supplementary material for: A multisectoral approach to developing a national strategy for mental health and suicide prevention in Sweden
Source: Glob Health Action. 2026 Sep 11;19(1):2679336. doi: 10.1080/16549716.2026.2679336 (PMC13573514; doi:10.1080/16549716.2026.2679336)
Supplement: Supplementary file 2 [file ZGHA_A_2679336_SM6979.docx]

**Table 1**: Overview of Mental Health and Suicide Prevention Policy Making in Sweden

| **Name of Policy/ Reform** | **Swedish Name** | **Time Period** | **Summary of Policy/ Reform Content** |
| --- | --- | --- | --- |
| Psychiatry Reform [9] | Psykiatrireformen | 1995 | The psychiatry reform aimed at shifting the focus of mental health care in Sweden from institutional care to community-based care and was directed at severely mentally ill people. The reform included various measures, such as developing community mental health centres, increasing access to outpatient care, and strengthening patient rights. |
| National Action Plan for the Development of Health Care [57] | Nationell handlingsplan för utveckling av hälso- och sjukvården | 2001-2007 | This national action plan aimed at improving the quality, accessibility, and efficiency of (mental) healthcare more broadly. The plan included various measures, such as increasing the availability of healthcare professionals, improving coordination between different levels of healthcare, and promoting evidence-based practices. |
| National Psychiatry Coordination [58] | Nationell psykiatrisamordning | 2003-2006 | The psychiatry coordination aimed at improving the quality and accessibility of mental health care in Sweden. Key measures were the promotion of collaboration and coordination between different agencies in the mental health field. |
| State Psychiatry Initiative [59] | Ambition och ansvar Nationell strategi för utveckling av samhällets insatser till personer med psykiska sjukdomar och funktionshinder | 2007-2011 | The initiative aimed at improving the quality of mental health care in Sweden by promoting evidence-based practices, increasing access to mental health care, and improving the coordination between different levels of mental health care. |
| National Action Program for Suicide Prevention [60] | Nationellt handlingsprogram för att förebygga självmord | 2008-current | The policy, adopted by parliament, aims to reduce the number of suicides in Sweden. A special focus lies on evidence-based methods, and various measures are included in the program, such as increasing the skills and knowledge of healthcare professionals, improving coordination between social services and healthcare, and promoting research in suicide prevention. |
| Mental Health Act [61] | Lag (1991:1128) om psykiatrisk tvångsvård | 2008 | The mental health act updated the former mental health legislation in Sweden and aimed at strengthening the rights of people with mental illness. The law outlines specific rights of people with mental illness, such as their right to informed consent, the right to be adequately informed about one’s condition and treatment, and a patient's right to have a say in their treatment decisions. |
| Plan for Targeted Intervention in the Field of Mental Illness (PRIO) [62] | Plan för riktade insatser inom området psykisk ohälsa (PRIO) | 2012-2015 | The plan aimed at improving the quality and accessibility of mental health care in Sweden through targeted interventions which focused on vulnerable groups, such as children and young people, people with substance abuse disorders, and people suffering from severe mental illness. The plan outlined various measures, such as increasing the availability of treatment, improving intersectoral coordination, and promoting digital technologies in mental health care. |
| Government's Strategy for Mental Health [10] | Regeringens strategi inom området psykisk ohälsa | 2016-2020 | The strategy for mental health aimed at reducing the prevalence of mental illness, improving access to care, and increasing the quality of care. The strategy outlined five focus areas and included six conditions to prevent mental illness better and support good mental health, such as improving cooperation within many parts of society. |

**Table 2**: Participant information

| **Governmental agency/ Organisation** | **Number of participants** | **Agency’s/ organisation’s policy involvement** | **Participants role and seniority** |
| --- | --- | --- | --- |
| Swedish Work Environment Authority | 1 | Core group of governmental agencies to collaborate on the strategy proposal development | Senior primary project respondent |
| Public Health Agency of Sweden | 2 | Instructed by the Swedish government to lead the work on the strategy proposal | Senior project respondents |
| Swedish Social Insurance Agency | 1 | Core group of governmental agencies to collaborate on the strategy proposal development | Senior primary project respondent |
| Swedish Gender Equality Agency | 1 | Core group of governmental agencies to collaborate on the strategy proposal development | Senior primary project respondent |
| Swedish Medical Products Agency | 1 | Core group of governmental agencies to collaborate on the strategy proposal development | Senior primary project respondent |
| Swedish Migration Agency | 1 | Core group of governmental agencies to collaborate on the strategy proposal development | Senior primary project respondent |
| Swedish Agency for Work Environment Expertise | 1 | Core group of governmental agencies to collaborate on the strategy proposal development | Senior primary project respondent |
| Swedish Agency for Participation | 1 | Core group of governmental agencies to collaborate on the strategy proposal development | Senior primary project respondent |
| Swedish Civil Contingencies Agency | 1 | Core group of governmental agencies to collaborate on the strategy proposal development | Senior primary project respondent |
| Swedish Agency for Youth and Civil Society | 1 | Core group of governmental agencies to collaborate on the strategy proposal development | Senior primary project respondent |
| Swedish Police | 1 | Core group of governmental agencies to collaborate on the strategy proposal development | Senior primary project respondent |
| Sámi Parliament of Sweden | 1 | Core group of governmental agencies to collaborate on the strategy proposal development | Junior primary project respondent |
| National Board of Health and Welfare | 1 | Instructed by the Swedish government to lead the work on the strategy proposal | Senior primary project respondent |
| Swedish Agency for Health Technology Assessment and Assessment of Social Services | 1 | Core group of governmental agencies to collaborate on the strategy proposal development | Senior primary project respondent |
| Swedish National Board of Institutional Care | 1 | Core group of governmental agencies to collaborate on the strategy proposal development | Senior primary project respondent |
| Swedish Media Council | 1 | Core group of governmental agencies to collaborate on the strategy proposal development | Senior primary project respondent |
| Swedish National Agency for Education | 1 | Core group of governmental agencies to collaborate on the strategy proposal development | Senior primary project respondent |
| Swedish Transport Administration | 3 | Core group of governmental agencies to collaborate on the strategy proposal development | Senior project respondents |
| Swedish Research Council | 1 | Core group of governmental agencies to collaborate on the strategy proposal development | Senior primary project respondent |
| Swedish Agency for Innovation Systems | 1 | Core group of governmental agencies to collaborate on the strategy proposal development | Senior primary project respondent |
| Suicide Zero | 1 | NGO which was consulted by the leading governmental agencies as an external stakeholder to provide input to the strategy proposal | Senior correspondent |
| Swedish Association of Local Authorities and Regions (SALAR) | 1 | Member-based organisation which was consulted by the leading governmental agencies as an external stakeholder to provide input to the strategy proposal | Senior correspondent |

**Interview Guide – National Authorities**

**Introductory Questions**

1. Could you please tell me a bit about your agency and your specific position?

2. How have you personally been involved in the development of the Swedish mental health and suicide prevention strategy?

**Process**

3. **Could you please tell me a bit about your perceptions of the process of the development of the current strategy?**

4. What has been done so far?

5. What did your agency (in particular) do so far?

6. What is the current state of action?

7. What will still be done before the new action plan needs to be presented in September? (From sides of your agency and in collaboration with the other agencies?)

8. What challenges emerged, and how did you overcome them?

**Actors**

9. Could you tell me a bit about your reflections on the actors involved in this task?

10. How did the collaboration with other involved agencies/ stakeholders go so far? a) What worked/ works great?
b) What could be improved for future collaboration?

**Content**

11. **What are your perceptions of the content of the new strategy?**

12. What do you see as the main strengths?

13. What do you see as challenges?

14. What has been (in-)sufficiently addressed?

**Context**

15. **What are your perceptions of the contextual factors that led to the mandate for a new mental health and suicide prevention strategy?**

16. What contextual factors do you believe played a role in the governmental mandate?

17. How will the new strategy relate to previous/ existing policy documents in the area?

**Implementation**

18. How should the finished strategy be implemented?

**Policy Documents**

19. What documents would you deem most essential to understand the work on the current development of the Swedish mental health and suicide prevention action plan?

**Conclusion**

20. Is there something important that we have not talked about that you think I should be aware of?

**Interview Guide – External Stakeholder**

**Introductory Questions**

21. Could you please tell me a bit about your organisation?

22. What are your job responsibilities – what do you work on?

**Main Questions**

23. How have you, as an organisation, been involved in the development of the new Swedish mental health and suicide prevention strategy?

24. Do you have any more reflections on that that you would like to share?

25. How closely do you generally work with the Public Health agency/ other agencies (on a national level)?

26. Do you have any reflections on the current development of a mental health and suicide prevention strategy?

- E.g., considering the **actors** involved?

- Considering the **process** of the development?

- Considering the **contextual factors** that have led to the development of a new strategy?

- Considering the **content** of the new strategy?

27. What are your personal wishes/ reflections on how the finished strategy should be implemented?

**Conclusion**

28. Is there something important that we have not talked about that you think I should be aware of or you would like to share?
